# Combination Effects of *Bacillus subtilis* and Chito-Oligosaccharides on Antioxidant Function, Immune Function and Intestinal Barrier Function in Broilers

**DOI:** 10.3390/biology15141159

**Published:** 2026-07-15

**Authors:** Jie Ma, Yeqing Liu, Yahui Zhao, Aiqin Gao

**Affiliations:** College of Animal Science, Inner Mongolia Agricultural University, Hohhot 010018, China

**Keywords:** *Bacillus subtilis*, chito-oligosaccharides, broilers, antioxidant capacity, immune function, intestinal barrier function

## Abstract

This study aimed to investigate the regulatory effects of combined *Bacillus subtilis* (BS) and chito-oligosaccharides (COS) supplementation on growth performance, antioxidant function, immune function, and intestinal barrier function of broilers. A total of 288 one-day-old broilers were randomly assigned to six groups, each consisting of six replicates of eight broilers. The control group was fed a basal diet (CON group). The experimental groups were fed the basal diet supplemented with 5 × 10^9^ CFU/kg BS (BS1 group), 6 × 10^9^ CFU/kg BS (BS2 group), 125 mg/kg COS (COS group), 5 × 10^9^ CFU/kg BS + 125 mg/kg COS (MIX1 group) and 6 × 10^9^ CFU/kg BS + 125 mg/kg COS (MIX2 group). The 42-day experiment consisted of two periods: a starter period (days 1–21) and a grower period (days 22–42). BS, COS and their combinations increased BW at 21 days (*p* < 0.05). The BS2, COS, MIX1, and MIX2 groups improved the average daily gain (ADG) during days 1–21 (*p* < 0.05). COS significantly reduced the feed conversion ratio (FCR) over 42 days (*p* < 0.05). For antioxidant function, MIX2 decreased serum MDA, while MIX1 increased hepatic GSH content, GSH-Px activity, and T-AOC (*p* < 0.05). MIX1 upregulated hepatic *CAT*, *GPX*, *SOD*, and *HO-1* mRNA expression and jejunal *SOD* expression (*p* < 0.05). For immune function, BS1 decreased jejunal IL-1β and IL-4 and increased IL-10 (*p* < 0.05), whereas MIX1 increased serum and hepatic IL-1β and IL-4 (*p* < 0.05). BS1 showed the highest hepatic *IL-10* expression, while MIX1 upregulated *NFKB1* and *RELA* (*p* < 0.05). For intestinal barrier, COS and MIX1 upregulated *JAM-2*, and BS1, MIX1, and MIX2 increased *Occludin*, with MIX2 showing the highest *ZO-1* expression (*p* < 0.05). In conclusion, dietary supplementation with BS and COS, either alone or in combination, can improve the growth performance, antioxidant function, immune function, and intestinal barrier function of broilers to varying degrees, with evident dose dependency and tissue specificity.

## Simple Summary

Modern poultry farming needs safe and natural alternatives to antibiotics to keep chickens healthy and productive. This study investigated whether combining a beneficial bacterium *Bacillus subtilis* (BS) and chito-oligosaccharides (COS) could improve the health and growth of broiler chickens. The results showed that BS, COS, or their combination increased body weight gain, enhanced the broilers’ natural antioxidant defense systems, and regulated immune responses in a balanced manner. Importantly, the effects depended on the dose used and varied across different tissues such as the liver and intestine. These findings suggest that this carefully formulated combination has strong potential to serve as a safe, natural substitute for antibiotics in broiler chicken diets. By reducing reliance on antibiotics, this strategy could contribute to more sustainable poultry production and help address public health concerns related to antibiotic resistance.

## 1. Introduction

Chicken is one of the most popular meat products worldwide, playing a crucial role in meeting the nutritional demands of the global population [1,2]. Over the past few decades, the demand for chicken meat has steadily increased [3]. With the development of modern animal husbandry, intensive farming has gradually replaced traditional farming methods to address the growing market demand [4]. Intensive production improves efficiency but increases poultry’s vulnerability to environmental, nutritional, and microbial challenges, which, when coupled with poor management, can severely impair their health and productivity [5]. For a long time, antibiotics have been widely used as poultry feed additives to alleviate stress responses, enhance breeding efficiency, accelerate poultry growth, inhibit the proliferation of harmful bacteria and reduce the risk of diseases [6,7]. Consequently, an increasing number of countries and regions have begun to restrict or prohibit the addition of antibiotics in poultry feed [8]. Driven by the global ban on antibiotic growth promoters, studies on related alternative solutions have been extensively conducted, and research on green feed additives (such as plant extracts, probiotics, and prebiotics) has become a core focus on animal nutrition [9]. Research on probiotics as an alternative to antibiotics has attracted growing attention, and their benefits as feed additives in promoting livestock and poultry growth and preventing diseases have been increasingly recognized [10]. *Bacillus subtilis* (BS) is a Gram-positive aerobic bacterium and an ideal multifunctional probiotic which can potentially inhibit pathogen growth and promote nutrient absorption [11]. BS can produce various bioactive molecules, such as digestive enzymes and antimicrobial peptides [12]. A large number of studies have shown that dietary supplementation with BS can improve the growth performance, antioxidant capacity, and immune function of livestock and poultry, maintaining intestinal microbial balance [13,14,15]. Gong et al. [16] reported that dietary supplementation with 1 × 10^8^ colony-forming units (CFU)/kg BS increased the average daily gain (ADG) and final body weight of Ross 308 broilers. This effect was accompanied by an improvement in serum antioxidant capacity. Liu et al. [17] found that dietary addition of 5 × 10^8^ CFU/kg BS significantly improved the feed conversion ratio (FCR) of white-feathered broilers and enhanced the antioxidant capacity of serum and liver tissues. In addition, BS enhances broiler intestinal health by improving the villus height/crypt depth (VH/CD) ratio, upregulating the expression of tight junction (TJ) proteins and reducing pathogenic bacterial colonization in the cecum, thereby synergistically improving intestinal barrier integrity and microbiota homeostasis [18,19].

The International Scientific Association for Probiotics and Prebiotics (ISAPP) stated in 2017 that prebiotics are substrates selectively utilized by host microorganisms to provide a health benefit [20]. Chito-oligosaccharides (COS), a type of prebiotic produced by enzymatic or acid hydrolysis of chitosan or chitin, possess characteristics such as low molecular weight and high water solubility, as well as various biological functions including anti-inflammatory, antioxidant, and anti-apoptotic activities [21,22]. In mammals, COS has been shown to interact with pattern recognition receptors such as Toll-like receptors (TLRs), activating macrophages and natural killer cells and promoting the secretion of cytokines, including interferon-γ (IFN-γ) and interleukin-2 (IL-2), thereby coordinating innate and adaptive immune responses [23,24]. Lan et al. [25] reported that COS can alleviate heat stress-induced abdominal fat deposition in broilers by enhancing hepatic antioxidant capacity and inhibiting inflammatory responses. Interestingly, COS can also mitigate liver injury, oxidative stress, and inflammatory responses in cold-stressed broilers, while reducing serum triglyceride (TG) and total cholesterol (TC) levels [26]. Furthermore, Gao et al. [27] found that dietary supplementation with 0.3% COS promoted the growth of mandarin fish by regulating the intestinal microbiota, activating the hepatic MAPK-NF-κB signaling pathway via the gut-liver axis, maintaining immune cell survival under inflammatory conditions and increasing intestinal immune substance levels. Consequently, a healthy intestinal barrier is essential for the efficient utilization of dietary nutrients, maintenance of normal growth and defense against pathogen invasion [28].

Currently, most studies have focused on the individual application of BS or COS, while their combined application and the resulting combination effects in livestock and poultry production have not been systematically investigated. Mechanistically, BS as a probiotic can consume intestinal oxygen, secrete digestive enzymes, and competitively inhibit pathogens, whereas COS as a prebiotic not only provides selective fermentation substrates for BS to promote its proliferation, but also directly modulates immune responses and intestinal barrier function. Therefore, the combined use of BS and COS may produce complementary and combination effects superior to those achieved by either alone. The present study aimed to evaluate the combined effects of dietary co-supplementation with BS and COS on growth performance, antioxidant capacity, immune function, and intestinal barrier function in broiler chickens, thereby providing experimental evidence for the scientific combination and application of probiotics and prebiotics in livestock and poultry production. These findings may also lay a foundation for the development of more effective synbiotic formulations and facilitate the practical application of combined probiotic–prebiotic strategies in the poultry industry.

## 2. Materials and Methods

The Animal Welfare and Ethics Committee of Inner Mongolia Agricultural University approved all animal experimental protocols. All procedures were performed in accordance with the approved guidelines.

### 2.1. Feed Additives

BS was supplied by Beihai Yiqiang Biotechnology Co., Ltd. (Beihai, China) with a viable count of 1 × 10^11^ CFU/g. COS was supplied by Shaanxi Niufu Biotechnology Co., Ltd. (Xi’an, China), with a purity of ≥98%, molecular weight < 1500 Da, and degree of deacetylation of ≥90%. Both BS and COS were used in powder form in this study.

### 2.2. Animals and Experimental Design

The experiment used 288 one-day-old white-feathered broilers of similar initial weight (46 ± 0.64 g), which were randomly divided into six groups. Each group contained six replicates, and each replicate housed eight broilers (equal numbers of males and females). Broilers in the control group were fed a basal diet (CON group). The experimental groups were fed the basal diet supplemented with 5 × 10^9^ CFU/kg BS (BS1 group), 6 × 10^9^ CFU/kg BS (BS2 group), 125 mg/kg COS (COS group), 5 × 10^9^ CFU/kg BS + 125 mg/kg COS (MIX1 group) and 6 × 10^9^ CFU/kg BS + 125 mg/kg COS (MIX2 group). Both BS and COS were first premixed with a small amount of the basal diet and then thoroughly mixed into the remaining feed to achieve the target inclusion levels. The basal diet was formulated in accordance with the *Feeding Standard of Chickens* (NY/T33-2004) [29], and its composition and nutritional levels are presented in Table 1. The basal diet consisted of a standard commercial formula feed provided by the experimental farm, in which cottonseed meal and rapeseed meal were included as conventional protein sources, resulting in a crude fiber level of approximately 7%. Although this fiber level is higher than that typically found in purified or semi-purified diets, it was intended to realistically simulate the practical production conditions under which the additives would be applied. All broilers were reared at the Integrated Broiler Production Base of the College of Animal Science, Inner Mongolia Agricultural University. Room temperature was initially set at 33 °C for the first week and then gradually reduced by 3 °C each week until reaching a constant temperature of 21 °C. Relative humidity was maintained at approximately 60%. The 42-day experiment consisted of two periods: a starter period (days 1–21) and a grower period (days 22–42). During the experiment, all broilers had free access to feed and water.

### 2.3. Sample Collection

Feed offered and refusals were recorded daily over the entire trial. On days 21 and 42, broilers were weighed individually following a 12 h fasting period (water provided ad libitum) to calculate ADG, average daily feed intake (ADFI), and FCR.

On day 42, after weighing, one bird per replicate whose body weight (BW) was nearest to the replicate mean was chosen for blood and tissue sampling. All sampling and preservation procedures followed standard protocols. Briefly, approximately 3–5 mL of blood was collected from the wing vein. After clotting, the blood was centrifuged at 3000× *g* for 15 min at 4 °C. Serum was stored at −80 °C until index measurement.

Following blood collection, the selected broilers were anesthetized and then euthanized by exsanguination. Tissue samples from the liver, jejunum, and jejunal mucosa were collected, immediately frozen in liquid nitrogen, and finally stored at −80 °C for the determination of antioxidant capacity and immune function-related indices. Specifically, a 3–4 cm segment of the mid-jejunum was excised. The mucosa was scraped from the jejunum with a sterile scalpel, and the leftover tissue was also saved.

### 2.4. Serum, Liver and Jejunum Antioxidant Indices

Using commercial kits from Nanjing Jiancheng Bioengineering Institute (Nanjing, China), we determined serum levels of total superoxide dismutase (T-SOD), total antioxidant capacity (T-AOC), malondialdehyde (MDA), catalase (CAT), and glutathione peroxidase (GSH-Px).

For tissue antioxidant analysis, 0.5 g of liver and 0.5 g of jejunum samples were separately placed in cryotubes and homogenized with 4.5 mL of normal saline to prepare a 1:9 (*w*/*v*) tissue homogenate. The homogenate was centrifuged at 3500 rpm for 15 min. The resulting supernatant was collected and stored for subsequent measurement. Tissue homogenates were stored at −80 °C. The antioxidant parameters in serum, liver, and jejunum were all determined in strict accordance with the manufacturer’s protocols.

### 2.5. Serum, Liver and Jejunum Immune Indices

Commercial ELISA kits obtained from Nanjing Jiancheng Bioengineering Institute (Nanjing, China) were used to assay immune indices in serum, liver, and jejunum. The immune indices measured included IFN-γ, IL-1β, IL-2, IL-4, IL-6, IL-10, TNF-α, and sIgA. Every procedure strictly adhered to the manufacturer’s protocols.

### 2.6. RNA Extraction, cDNA Synthesis and Quantitative Real-Time PCR

Total RNA was isolated using the RNAiso Plus Kit (TaKaRa, Beijing, China) as per the manufacturer’s instructions. Briefly, 50 mg samples of liver, jejunum tissue, and jejunal mucosa were weighed and lysed with 1 mL of TRIzol reagent. Mechanical homogenization was performed using an FA-25D homogenizer (Fluko Technology Development Co., Ltd., Shanghai, China). Total RNA was isolated following the method described by Ji et al. [30]. RNA concentration and purity (OD260/280 and OD260/230 ratios) were assessed on an Implen P330 spectrophotometer (Munich, Germany).

Using the Evo M-MLV Reverse Transcription Kit (Accurate Biotechnology, Changsha, China), 2 μL of RNA (500 ng/μL) was mixed with 2 μL of gDNA Clean Reaction Mix and 6 μL of RNase-free water, incubated in a LabCycler (SensoQuest, Göttingen, Germany) for 2 min, and then supplemented with 4 μL of 5× Evo M-MLV RT Reaction Mix and another 6 μL of RNase-free water. The mixture was heated at 37 °C for 15 min, 85 °C for 5 s, and finally chilled to 4 °C.

qPCR was performed with SYBR Green Pro Taq HS qPCR Kit (Accurate Biotechnology, Changsha, China) on a LightCycler 480 II (Roche, Basel, Switzerland). A 20-μL mixture (2 μL cDNA, 0.5 μL each primer, 10 μL 2× Premix, 7.2 μL RNase-free water) was subjected to 95 °C for 30 s, then 40 cycles of 95 °C for 5 s and 60 °C for 30 s.

β-Actin was used as the internal reference gene for data normalization. The relative mRNA expression levels of target genes, including *Nrf2*, *GPX*, *CAT*, *SOD*, *HO-1*, *IL-1β*, *IL-6*, *IL-10*, *TLR4*, *MyD88*, *NFKB1*, *RELA*, *Occludin*, *ZO-1*, *JAM2* and *MUC2*, were determined. β-Actin was used as the internal reference gene for data normalization, the 2^−ΔΔCt^ method was used to calculate the relative mRNA expression of target genes [31]. Specific primers for antioxidant-, immune-, and intestinal barrier-related genes were created using Primer-BLAST (NCBI; www.ncbi.nlm.nih.gov. accessed on 20 April 2026), and specificity was validated. All primer sequences are shown in Table 2.

### 2.7. Data Analysis

Prior to statistical analysis, all data were subjected to the Shapiro–Wilk test for normality and Levene’s test for homogeneity of variance, data with *p* > 0.05 were considered to be normally distributed [32]. One-way ANOVA was employed to examine overall differences among the six groups. Subsequent pairwise comparisons were made using the Tukey–Kramer post hoc procedure. All analyses were carried out with SPSS version 26.0 (IBM, Armonk, NY, USA), and the level of significance was set at *p* < 0.05.

## 3. Results

### 3.1. Effects of BS and COS Supplementation on Growth Performance of Broilers

The growth performance of broilers in response to dietary BS and COS supplementation is summarized in Table 3. Compared with the CON group, the BS2, COS, MIX1, and MIX2 groups all showed significantly higher BW on day 21 (*p* < 0.05), with the MIX1 group exhibiting the greatest increase (+4.3%); however, no significant differences were observed among the experimental groups. On day 42, BW did not differ significantly among any groups. For ADG during days 1–21, the BS2, COS, MIX1, and MIX2 groups were significantly higher than the CON group (*p* < 0.05), with the MIX1 group showing a 4.5% increase, but no significant differences were found among the experimental groups. No significant differences in ADG were detected during days 22–42 or days 1–42.

Regarding ADFI during days 1–21, the BS1, BS2, COS, and MIX1 groups were significantly higher than the CON group (*p* < 0.05), whereas the MIX2 group showed no difference from CON and was significantly lower than all other experimental groups (*p* < 0.05). During days 22–42, ADFI in the BS1, COS, and MIX1 groups was significantly higher than that in the CON group (*p* < 0.05), and the MIX1 group had significantly higher ADFI than the BS2 and MIX2 groups (*p* < 0.05). Over the entire period (days 1–42), the COS and MIX1 groups had significantly higher ADFI than the CON group (*p* < 0.05), and the MIX1 group was significantly higher than the BS1, BS2, and MIX2 groups (*p* < 0.05). For FCR during days 1–21, the MIX2 group was significantly higher than the CON and all other experimental groups (*p* < 0.05), while the BS1 group was significantly lower than the MIX2 group (*p* < 0.05). No significant differences in FCR were observed during days 22–42. During days 1–42, the COS group had significantly lower FCR than the CON group (*p* < 0.05), and the MIX2 group had significantly higher FCR than the CON, COS, and MIX1 groups (*p* < 0.05). These results indicate that MIX1 exhibited the best early growth performance, though not significantly superior to the individual additive groups; MIX2, despite controlling feed intake, showed a significantly poorer FCR; and COS alone most effectively improved overall feed efficiency.

### 3.2. Effects of BS and COS Supplementation on Antioxidant Function in the Serum, Liver, and Jejunum of Broilers

#### 3.2.1. Effects of BS and COS Supplementation on Antioxidant Capacity in Serum, Liver and Jejunum of Broilers

The effects of BS and COS supplementation on serum antioxidant capacity of broilers are shown in Table 4. Compared with the CON group, serum MDA content in the MIX2 group was significantly decreased (*p* < 0.05), while no significant differences were observed in the BS1, BS2, COS, or MIX1 groups. No significant differences among groups were found for CAT, SOD, GSH, GSH-Px or T-AOC (*p* > 0.05).

The effects of supplementation with BS and COS on hepatic antioxidant capacity of broilers are presented in Table 5. Serum SOD activity remained unaffected by dietary treatments, with no significant differences detected among groups (*p* > 0.05). In contrast, the BS1 group showed a significant reduction in CAT activity, GSH content, GSH-Px activity, and T-AOC relative to the CON group (*p* < 0.05). MDA content was also significantly lower in the BS1 group (*p* < 0.05). Notably, MIX1 significantly elevated GSH content compared with the CON group (*p* < 0.05). For these indices, no other experimental groups differed from the CON group (*p* > 0.05).

The effects of BS and COS supplementation on jejunal antioxidant capacity of broilers are summarized in Table 6. No significant differences in CAT, SOD, GSH, GSH-Px, T-AOC or MDA were observed between the CON group and any of the experimental groups (*p* > 0.05).

#### 3.2.2. Effects of Dietary BS and COS Supplementation on Antioxidant Gene Expression in the Liver and Jejunum of Broilers

The effects of BS and COS supplementation on the expression of hepatic antioxidant-related genes in broilers are presented in Table 7. *CAT* expression was significantly upregulated in all treatment groups relative to the CON group (*p* < 0.05); among them, the BS1 group exhibited the highest level, which was significantly greater than that in the BS2, COS, and MIX2 groups (*p* < 0.05). *GPX* expression increased significantly in BS1, COS, and MIX1 compared with CON (*p* < 0.05). *Nrf2* expression was higher in BS1 and BS2 than in CON (*p* < 0.05). *SOD* expression was elevated in all treated groups versus CON (*p* < 0.05), with MIX1 showing significantly higher expression than all other treatment groups (*p* < 0.05). Similarly, *HO-1* expression was upregulated across all experimental groups (*p* < 0.05), and MIX1 was significantly higher than BS1, COS, and MIX2 (*p* < 0.05).

The effects of BS and COS supplementation on the expression of antioxidant-related genes in the jejunum of broilers are shown in Table 8. *CAT* mRNA expression differed significantly among groups (*p* < 0.05), with the BS2 group showing higher expression than all other groups. The CON and BS1 groups did not differ significantly, and both were lower than COS, MIX1, and MIX2 (*p* < 0.05). *GPX* expression was unaffected by dietary treatments (*p* > 0.05). *Nrf2* expression varied among groups (*p* < 0.05); BS1 and MIX1 had significantly greater expression than CON, COS, and MIX2 (*p* < 0.05). *SOD* expression also showed significant intergroup differences (*p* < 0.05). MIX1 exhibited the highest expression, while COS and MIX2 were significantly higher than CON, BS1, and BS2 (*p* < 0.05); no differences were detected among CON, BS1, and BS2. *HO-1* mRNA levels remained comparable across groups (*p* > 0.05).

### 3.3. Effects of BS and COS Supplementation on Immune Function in Serum, Liver and Jejunum of Broilers

#### 3.3.1. Effects of BS and COS Supplementation on Immune Indices in the Serum, Liver, and Jejunum of Broilers

The effects of BS and COS supplementation on serum immune indices of broilers are presented in Table 9. Serum IL-1β concentrations were elevated in the BS1, MIX1, and MIX2 groups relative to the CON group (*p* < 0.05). IL-4 levels rose significantly in BS1, BS2, MIX1, and MIX2 (*p* < 0.05), with MIX1 showing the highest numerical value, although the differences among these four groups did not reach statistical significance (*p* > 0.05). IL-10 content increased in BS1 and MIX2 (*p* < 0.05), whereas it was markedly lower in MIX1 compared with CON (*p* < 0.05). TNF-α was significantly decreased only in the MIX1 group (*p* < 0.05), and its level was lower than that of all other experimental groups (*p* < 0.05). No significant alterations were detected for IFN-γ, IL-2, or IL-6 across groups (*p* > 0.05).

The effects of BS and COS supplementation on hepatic immune indices of broilers are presented in Table 10. IL-1β levels were significantly higher in the BS1, COS, MIX1, and MIX2 groups than in the CON group (*p* < 0.05); among these, BS1 was significantly higher than BS2 and MIX2 (*p* < 0.05). IL-2 concentration was significantly decreased only in BS1 (*p* < 0.05). IL-4 levels were elevated in BS1, COS, MIX1, and MIX2 versus CON (*p* < 0.05), with BS1 exceeding all other treatment groups (*p* < 0.05). IL-6 was significantly lower exclusively in BS1 (*p* < 0.05). No significant changes in IFN-γ, IL-10, or TNF-α were detected among the groups (*p* > 0.05).

The effects of BS and COS supplementation on jejunal immune indices of broilers are shown in Table 11. Significant intergroup differences were detected for IL-1β, IL-2, IL-4, IL-6, IL-10, and TNF-α (*p* < 0.05). Both IL-1β and IL-4 levels were markedly lower in the BS1 group than in all other groups (*p* < 0.05). In contrast, IL-2, IL-6, and TNF-α concentrations in the CON group were significantly lower than those in the BS1, COS, MIX1, and MIX2 groups (*p* < 0.05), with the exception of IL-10, where CON was lower than BS1, COS, and MIX1 (*p* < 0.05). No significant changes were observed for IFN-γ or sIgA (*p* > 0.05).

#### 3.3.2. Effects of BS and COS Supplementation on Immune Gene Expression in the Liver and Jejunum of Broilers

The effects of BS and COS supplementation on the expression of hepatic immune-related genes in broilers are presented in Table 12. *IL-1β* mRNA expression showed significant intergroup variation (*p* < 0.05), with the BS2, CON, and MIX1 groups exhibiting significantly higher levels than the COS and MIX2 groups (*p* < 0.05). *IL-6* expression was significantly elevated in the BS2 and MIX1 groups compared with the CON, COS, and MIX2 groups (*p* < 0.05). For *IL-10*, a significant treatment effect was detected (*p* < 0.05): BS1 and BS2 displayed the highest expression, with no statistical difference between them; COS and MIX2 were similar to each other and were significantly higher than CON and MIX1, yet significantly lower than BS1 and BS2 (*p* < 0.05). *TLR4* expression did not differ significantly among the groups (*p* > 0.05). *MyD88* expression was significantly influenced by treatment (*p* < 0.05), with BS2 and COS showing significantly higher levels than BS1 and MIX1 *(p* < 0.05). *NFKB1* expression differed significantly among groups (*p* < 0.05), and MIX1 was significantly higher than CON, BS1, and BS2 (*p* < 0.05). *RELA* expression also varied significantly (*p* < 0.05): MIX1 attained the highest expression; BS2 was significantly higher than CON, BS1, COS, and MIX2, but significantly lower than MIX1; COS and MIX2 were statistically indistinguishable and were significantly lower than both BS2 and MIX1 (*p* < 0.05).

The effects of BS and COS supplementation on the expression of jejunal immune-related genes in broilers are summarized in Table 13. *IL-1β* mRNA expression was similar among the BS2, CON, and MIX1 groups, and all three were significantly higher than COS and MIX2 (*p* < 0.05). *IL-6* expression was significantly elevated in BS2 and MIX1 compared with CON, COS, and MIX2 (*p* < 0.05). *IL-10* showed an overall treatment effect (*p* < 0.05), with the highest expression in BS1 and BS2, which did not differ significantly from each other. *TLR4* expression did not change significantly among groups (*p* > 0.05). *MyD88* expression exhibited a significant overall effect (*p* < 0.05), as BS2 and COS were significantly higher than BS1 and MIX1 (*p* < 0.05). *NFKB1* varied significantly among treatments (*p* < 0.05), with MIX1 being significantly higher than CON, BS1, and BS2 (*p* < 0.05). *RELA* also showed significant intergroup differences (*p* < 0.05); MIX1 displayed the highest expression level.

### 3.4. Effects of BS and COS Supplementation on Intestinal Barrier Function of Broilers

The effects of BS and COS supplementation on the intestinal barrier function of broilers are summarized in Table 14. A significant treatment effect was detected for *JAM2* expression (*p* < 0.05): COS and MIX1 showed the highest levels and did not differ from each other, whereas MIX2 exhibited the lowest expression, significantly lower than all other groups (*p* < 0.05). *Occludin* expression also varied among groups (*p* < 0.05). MIX2 displayed the highest level; BS1 and MIX1 were intermediate, with no statistical difference between them, and both were significantly higher than CON, BS2, and COS (*p* < 0.05). *ZO-1* expression was significantly affected by treatment (*p* < 0.05), with COS being significantly higher than every other group (*p* < 0.05). No significant variation was observed for *MUC2* expression (*p* > 0.05).

## 4. Discussion

### 4.1. Effects of Dietary BS and COS Supplementation on Growth Performance of Broilers

Bozkurt et al. reported that simultaneous supplementation of prebiotics and probiotics significantly improved FCR, indicating a combination effect between the two supplements [33]. In the present study, dietary supplementation with BS and COS, alone or in combination, increased ADFI, ADG, and BW at 21 days of age. However, the effects on FCR varied among treatments: the MIX2 group showed increased FCR during 1–21 days, whereas the COS group exhibited decreased FCR over the entire period of 1–42 days. No significant differences in growth performance parameters were observed during days 22–42 or in BW at 42 days of age. These results indicate that dietary supplementation with BS and COS can improve the early growth of broilers, which is in partial agreement with earlier reports. Khongthong et al. demonstrated that dietary supplementation with 1 × 10^6^ CFU/g BS or 1 × 10^7^ CFU/g BS improved BW in broilers (*p* < 0.05) [34]. Lan et al. also found that COS supplementation improved ADG and ADFI in heat-stressed yellow-feathered broilers aged 57–77 days [35]. The improvement in ADG may be attributed to enhanced nutrient digestibility, intestinal barrier function, antioxidant capacity, and immune function [36,37,38]. It appears that stage-specific optimization of dietary BS and COS could better align with the changing nutritional demands of broilers.

### 4.2. Effects of Dietary BS and COS Supplementation on Antioxidant Function in Serum, Liver and Jejunum of Broilers

#### 4.2.1. Effects of Dietary BS and COS Supplementation on Antioxidant Capacity in Serum, Liver and Jejunum of Broilers

Oxidative stress is a pathological state arising from an imbalance between reactive oxygen species (ROS) production and antioxidant defense, leading to excessive ROS accumulation and subsequent damage to cells and tissues [39]. Enzymatic antioxidants—CAT, SOD and GSH-Px—convert ROS into harmless substances like water and oxygen, thus maintaining redox balance and protecting the organism [40]. Free radicals damage membrane lipids, generating MDA, which further propagates lipid peroxidation and exacerbates oxidative stress; T-AOC is commonly used to evaluate overall antioxidant potential [41]. In the present study, serum MDA content was significantly decreased in the MIX2 group (*p* < 0.05), and hepatic GSH content, GPX activity, and T-AOC were significantly increased in the MIX1 group (*p* < 0.05). However, the remaining antioxidant parameters in serum, liver, and jejunum did not differ significantly among groups. These results suggest that the effects of BS and COS on antioxidant capacity are tissue-specific and dose-dependent. Although some indices exhibited numerical trends (e.g., a slight decrease in hepatic MDA in the BS1 group and a slight increase in jejunal GSH-Px activity in the MIX2 group), these differences did not reach statistical significance; therefore, definitive conclusions cannot be drawn. This may be related to factors such as supplementation dosage and duration of action. These results are partially consistent with previous studies. Liu et al. reported that dietary supplementation with 5 × 10^8^ CFU/kg BS enhanced serum and hepatic antioxidant capacity and reduced diamine oxidase activity and endotoxin levels in broilers (*p* < 0.05) [17]. Another study demonstrated that dietary COS supplementation decreased serum and hepatic MDA levels, and increased serum SOD and CAT activities as well as hepatic GSH-Px activity in heat-stressed broilers, suggesting that COS may alleviate oxidative damage induced by high-temperature stress [25].

#### 4.2.2. Effects of Dietary BS and COS Supplementation on Antioxidant Gene Expression in the Liver and Jejunum of Broilers

*Nrf2* is a key transcription factor in the antioxidant system that plays an indispensable role in defending against oxidative stress by regulating the expression of antioxidant enzyme-related genes [42]. Under oxidative stress conditions, ROS can disrupt the binding between *Nrf2* and Kelch-like ECH-associated protein 1 (*Keap1*) through the modification of specific cysteine residues, thereby facilitating the translocation of *Nrf2* into the nucleus, where it forms a heterodimer with small MAF proteins [43]. These heterodimers can bind to specific DNA sequences, such as antioxidant response elements (AREs), thereby further upregulating the expression of genes encoding phase II detoxifying enzymes, including NAD(P)H:quinone oxidoreductase 1 (*NQO1*) and *HO-1* [44].

The hepatic mRNA levels of *CAT*, *GPX*, *SOD*, *Nrf2*, and *HO-1* were markedly upregulated by dietary BS and COS in this study. In the MIX1 group, the hepatic expression of all these genes was significantly upregulated (*p* < 0.05). Jejunal *CAT*, *Nrf2*, and *SOD* mRNA levels were also significantly elevated in the MIX1 group (*p* < 0.05). The upregulation of these genes is partially consistent with previous studies. For instance, Zhang et al. reported that BS enhanced antioxidant capacity by upregulating the expression of *Nrf2*, *HO-1*, and *GPX* in the liver [45], and Lan et al. demonstrated that COS alleviated oxidative damage induced by heat stress through activation of the Nrf2 pathway and upregulation of *CAT* mRNA expression [25]. However, other studies have reported that COS reduced *Nrf2* mRNA expression in the jejunum and ileum, as well as *HO-1* mRNA expression in the duodenum and ileum [46]. The stronger upregulation of antioxidant gene expression observed in the liver relative to the jejunum may reflect tissue-specific differences in metabolic demand and exposure to dietary components. As the central metabolic organ, the liver is highly responsive to systemic changes induced by BS and COS or their metabolites, whereas the jejunal mucosa, which directly contacts digesta, may require higher local concentrations or longer exposure to achieve a comparable transcriptional response. This tissue-specific pattern suggests that the threshold for transcriptional activation differs between liver and intestine. Moreover, the observed dose-dependent effects indicate that the current supplementation level is sufficient to initiate gene transcription but may be inadequate to overcome post-transcriptional regulatory barriers that limit the actual synthesis or activity of antioxidant enzymes. The lack of significant changes in serum and jejunal enzyme activities supports the notion that functional antioxidant enhancement at the protein level likely demands higher doses or a prolonged feeding period. Thus, both tissue specificity and dose dependency are critical factors that govern the ultimate antioxidant outcomes of BS and COS supplementation. Extended experimental durations or elevated dosages may be necessary to translate the transcriptional responses into measurable improvements in enzyme activity, a hypothesis that warrants further investigation.

An interesting observation in the present study was the discordance between hepatic *CAT* mRNA expression and antioxidant enzyme activities in the BS1 group, where upregulated CAT transcript levels were accompanied by decreased CAT activity, GSH-Px activity, and T-AOC in the liver. As the central metabolic organ, the liver is highly sensitive to dietary interventions and may exhibit a compensatory transcriptional response to increased oxidative challenge. The elevated *CAT* mRNA in the BS1 group might reflect such a mechanism—an attempt to counteract enhanced reactive oxygen species production induced by the specific dose of BS. However, the corresponding decline in enzyme activities suggests that the newly synthesized mRNA was not effectively translated into functional proteins. This could be due to limitations in hepatic translational capacity, insufficient availability of cofactors, or accelerated enzyme protein degradation and consumption under oxidative stress. Furthermore, post-translational modifications or direct interference by BS-derived metabolites with enzyme assembly in hepatocytes may also contribute to the observed inconsistency. These results highlight that, in the liver, transcriptional activation alone does not guarantee enhanced antioxidant capacity, and the net functional outcome depends on the balance between enzyme synthesis, consumption, and post-transcriptional regulatory processes.

### 4.3. Effects of Dietary BS and COS Supplementation on Immune Function in Serum, Liver and Jejunum of Broilers

#### 4.3.1. Effects of Dietary BS and COS Supplementation on Immune Indices in the Serum, Liver, and Jejunum of Broilers

Inflammatory cytokines play crucial roles in orchestrating immune and inflammatory responses, and dysregulated inflammation is a key hallmark of intestinal dysfunction [47]. Cytokines are small-molecule signaling proteins secreted by immune cells upon stimulation, mediating defense against foreign pathogens [48]. In the present study, dietary BS and COS supplementation exerted tissue-specific effects on immune indices in broilers. In serum, IL-1β was increased in the BS1, MIX1, and MIX2 groups, and IL-4 was increased in the BS1, BS2, MIX1, and MIX2 groups. IL-10 was higher in the BS1 and MIX2 groups but lower in the MIX1 group, and TNF-α was reduced only in the MIX1 group. In the liver, IL-1β and IL-4 were broadly upregulated in the BS1, COS, and MIX groups, whereas IL-2 and IL-6 were decreased exclusively in the BS1 group. In the jejunum, the response pattern differed markedly, IL-1β and IL-4 were decreased in the BS1 group, while IL-2, IL-6, IL-10, and TNF-α were generally upregulated in the BS1, COS, and MIX groups. These findings indicate that BS and COS exert a complex immunomodulatory action on both pro- and anti-inflammatory cytokines in a highly tissue- and dose-dependent manner, with the BS1 treatment often showing distinct or even opposite effects compared with other groups. Importantly, while the upregulation of IL-4 may reflect a Th2-skewing or anti-inflammatory potential, the concurrent elevation of IL-1β—a classical pro-inflammatory cytokine—in serum and liver suggests that BS and COS do not simply suppress or enhance immunity but rather prime or recalibrate the immune system. Whether such cytokine changes ultimately confer protection or pose a risk of excessive inflammation likely depends on the balance between pro- and anti-inflammatory signals, the specific tissue context, and the duration of intervention. Therefore, these cytokine alterations should not be simply interpreted as beneficial; rather, they highlight the nuanced immunomodulatory properties of these additives and caution against generalizing their effects across different immune compartments. Previous studies have reported related findings. Liu et al. demonstrated that dietary BS supplementation increased serum IL-6 levels in broilers at 21 days of age, decreased serum TNF-α levels at 50 days of age, and upregulated the mRNA expression of IL-10, IL-22, and IFN-γ in the jejunal mucosa, suggesting that BS may serve as a promising immunomodulator for intestinal immune function in broilers [17]. Lan et al. found that dietary COS supplementation significantly reduced serum IL-1β and TNF-α levels, significantly increased serum IL-10 levels and tended to significantly elevate IL-10 levels in the liver and spleen (*p* < 0.05) [35]. The discrepancies between these studies and our findings may be attributed to differences in experimental models, dosage regimens, or sampling time points, further underscoring the context-dependent nature of the immunomodulatory effects of BS and COS.

sIgA inhibits the adhesion and invasion of potentially harmful antigens to the intestinal mucosa, neutralizes toxins and virulence factors produced by microbial pathogens, and thus protects the intestine from dietary and microbial antigens [49]. In the present study, dietary supplementation with BS and COS numerically increased jejunal sIgA content, although this change did not reach statistical significance. This numerical trend suggests that, at the current dosage, BS and COS may have initiated a regulatory response that enhances intestinal mucosal immunity, but the effect has not yet reached a detectable magnitude. This observation is consistent with previous research. Rajput et al. reported that dietary BS significantly enhanced sIgA secretion in the jejunum and ileum of broilers [50], while Xiong et al. demonstrated that COS supplementation increased sIgA contents in the duodenum and ileum of weaned piglets [51]. The lack of statistical significance in our study may be due to the relatively short experimental period or the moderate supplementation dosage, and more pronounced effects might be achieved with prolonged administration or higher inclusion levels.

#### 4.3.2. Effects of Dietary BS and COS Supplementation on Immune Gene Expression in the Liver and Jejunum of Broilers

The TLR signaling pathway activates NF-κB through IκB phosphorylation, thereby triggering cytokine production [52]. MyD88 serves as an adaptor protein for the downstream inflammatory signaling pathways of all TLRs except TLR3; the MyD88 signaling pathway stimulates the production of inflammatory cytokines by activating the transcription factor NF-κB [53]. Tobita reported that BS downregulated *TLR4* mRNA expression while upregulating *MyD88* and *NF-κB* mRNA expression [54]. Shi et al. demonstrated that COS inhibited LPS- and DSS-induced inflammatory responses in IPEC-J2 cells and mice via the TLR4/NF-κB signaling pathway [55]. The present results indicate that BS and COS supplementation can regulate immune-related genes in the TLR4/NF-κB signaling pathway, thereby contributing to the modulation of immune function and the balance between pro- and anti-inflammatory cytokines. In the present study, dietary BS and COS supplementation regulated immune-related gene expression in the liver and jejunum of broilers in a distinct dose- and tissue-dependent manner, with different treatment groups exerting different patterns of influence on various immune factors. Both the BS1 and BS2 groups significantly upregulated the anti-inflammatory cytokine *IL-10*. Notably, while promoting *IL-10* expression, the BS2 group also sustained relatively high expression of the pro-inflammatory cytokines *IL-1β* and *IL-6*, suggesting that the BS2 group may simultaneously activate both pro- and anti-inflammatory signals, thereby enabling the intestinal immune system to establish a state of enhanced immune surveillance that remains controlled—a state conducive to rapid responses against potential pathogens. In contrast, the COS and MIX2 groups exhibited overall low expression of *IL-1β* and *IL-6*, even lower than the CON group, whereas their *IL-10* expression was at an intermediate level. This indicates that the COS and MIX2 groups may be more inclined to suppress the basal transcription of pro-inflammatory cytokines, exhibiting an immunoregulatory state characterized by suppressed pro-inflammatory signaling. This observation is consistent with the findings of Xiong et al. [51], who reported that COS reduced intestinal inflammatory cytokine expression.

The unique performance of the MIX1 group deserves particular discussion. This group exhibited the highest expression of *NFKB1* and *RELA* in both the liver and jejunum, accompanied by high levels of IL-1β and IL-6, whereas its IL-10 level was significantly lower than those in the BS1, BS2, COS, and MIX2 groups. This pro-inflammatory dominant phenotype suggests that the combination of BS and COS in the MIX1 group may activate the NF-κB signaling pathway, thereby promoting the transcriptional initiation of immune-related genes. However, *TLR4* mRNA expression did not change significantly in any group, and only its downstream adaptor molecule *MyD88* was upregulated in the BS2 and COS groups. This indicates that the regulation of the NF-κB pathway by BS and COS may not depend on *TLR4* ligand recognition but rather may occur through modulation of intracellular adaptor proteins or direct effects on the expression of transcription factors—mechanisms that require further verification. Furthermore, integrating these immune gene expression results with the previous antioxidant indicators reveals a common phenomenon: in the MIX1 group, the hepatic mRNA levels of antioxidant genes (*CAT*, *GPX*, *SOD*, *Nrf2*, *HO-1*) and immune signaling molecules (*NFKB1*, *RELA*) were all significantly upregulated, yet the corresponding enzyme activities in serum and jejunum, as well as immune effector molecules such as sIgA, showed relatively limited improvement, with some not even reaching statistical significance. This further supports our speculation that, under the current dosage and treatment duration, the effects of BS and COS are primarily manifested as a “gene-priming” effect at the transcriptional level, while rate-limiting factors may exist in the translation and post-translational regulation from mRNA to functional proteins. Future studies incorporating extended intervention periods or moderately increased doses, combined with proteomic analyses, will help elucidate the complete regulatory network underlying this transcription–function discrepancy, thereby enabling a more precise assessment of the actual contributions of BS and COS to the immune and antioxidant functions of broilers.

### 4.4. Effects of Dietary BS and COS Supplementation on Intestinal Barrier Function of Broilers

Intestinal barrier function determines both the body’s resistance to external stimuli and its ability to maintain internal environmental homeostasis. The intestinal epithelial barrier prevents the penetration of macromolecules and pathogenic microorganisms, ensures nutrient absorption, and recognizes harmful microorganisms and antigens through various mechanisms [56,57]. Previous studies have shown that dietary BS supplementation can improve intestinal barrier integrity by regulating the intestinal microenvironment and associated microbial community composition [58], while COS can maintain the expression of TJ proteins and mucins by modulating cytokine secretion, thereby promoting intestinal barrier repair [59]. TJs are key components of the intestinal mucosal barrier and are responsible for regulating paracellular permeability [60]. They interact with cytoplasmic scaffold proteins, which in turn bind to the actin cytoskeleton [61]. Previous studies have demonstrated that BS supplementation significantly upregulates the mRNA expression levels of TJ proteins *JAM2*, *ZO-1*, and *Occludin* [62]. Wang et al. reported that COS alleviated dextran sulfate sodium (DSS)-induced mucus disruption by increasing MUC2 secretion and promoting *MUC2* mRNA expression in HT-29 cells [63]. Consistent with these findings, the present study demonstrated that dietary BS and COS supplementation maintained intestinal epithelial barrier function by upregulating the mRNA expression of *ZO-1*, *Occludin* and *JAM2* in the jejunum.

Supplementing broiler diets with BS and COS had differential effects on jejunal TJ gene expression. Relative to the CON group, *JAM2* mRNA was significantly upregulated in the COS and MIX1 groups, whereas it was sharply downregulated in the MIX2 group. *Occludin* expression was highest in the MIX2 group and was also significantly upregulated in the BS1 and MIX1 groups relative to the CON, BS2, and COS groups. *ZO-1* expression was significantly elevated only in the COS group. Both individual and combined BS and COS improved the expression of specific TJ proteins in a gene- and dose-dependent fashion, suggesting a potential beneficial effect on intestinal barrier integrity.

## 5. Conclusions

These findings demonstrate that broiler diets supplemented with BS, COS, or their combination can enhance growth, antioxidant defense, immune response, and intestinal barrier function to different extents, depending on dose and tissue type. These results not only provide a theoretical basis and practical reference for the rational combination of probiotics and prebiotics in broiler production, but also further support the potential of combined BS and COS as a natural alternative to antibiotic growth promoters.

## Figures and Tables

**Table 1:** Ingredient and nutrient composition of the basal diet (%, as-fed basis).

Items	1–21 Days	22–42 Days
Ingredients		
Corn	58.95	60.9
Soybean oil	3	3
Soybean meal	23	20
Cottonseed cake	6	6
Rape seed cake	5	6
Limestone	1.07	1.22
CaHPO_4_	1.9	1.8
NaCl	0.37	0.37
Choline	0.11	0.11
Vitamin premix ^1^	0.1	0.1
Mineral premix ^2^	0.5	0.5
Total	100	100
Nutrient levels		
ME (MJ/kg)	12.26	12.64
CP (%)	21.00	20.00
CF(%)	7.00	6.00
Ca (%)	1.05	0.95
P (%)	0.52	0.47
Met + Cys (%)	0.82	0.71

Note: ^1^ The Vitamin premix provides the following per kg of the diet: VA—9000 IU; VB1—3.00 mg, VB_2_—8.00 mg, VB_6_—4.40 mg, VB_12_—0.012 mg, VD_3_—3000 IU, VE—26 IU, VK_3_—1.20 mg, calcium pantothenate—1 mg, nicotinic acid—45 mg.; ^2^ The mineral premix provides the following elements per kg of the diet: Fe—100 mg, Cu—10 mg, Mn—120 mg, Zn—108 mg, I—1.50 mg, Se—0.35 mg. ME is calculated, and the rest are measured. Abbreviations: ME: metabolic energy; CP: crude protein; CF: crude fiber.

**Table 2:** Primer sequences for quantitative real-time PCR targeting specific genes.

Gene	Sequence (5′–3′)	GenBank No.
*β-* *actin*	F:5-GCCAACAGAGAGAAGATGACAC-3 R:5-GTAACACCATCACCAGAGTCCA-3	NM_205518
*Nrf2*	F:5-GATGTCACCCTGCCCTTAG-3 R:5-CTGCCACCATGTTATTCC-3	NM_205117.1
*GPX*	F:5-CAAAGTTGCGGTCAGTGGA-3 R:5-AGAGTCCCAGGCCTTTACTACTTTC-3	NM_001163245.1
*CAT*	F:5-GTTGGCGGTAGGAGTCTGGTCT-3 R:5-GTGGTCAAGGCATCTGGCTTCTG-3	NM_001031215.1
*SOD*	F:5-TTGTCTGATGGAGATCATGGCTTC-3 R:5-TGCTTGCCTTCAGGATTAAAGTGA-3	NM_205064.1
*HO-1*	F:5-GGTCCCGAATGAATGCCCTTG-3 R:5-ACCGTTCTCCTGGCTCTTGG-3	HM237181.1
*IL-10*	F:5-TTATTATCCAGGTGGCAGTCGA-3 R:5-CTTAGGTCGCGTTTCGG-3	NM_001004414
*IL-1β*	F:5-CAGCCTCAGCGAAGAGACCT-3 R:5-ACTGTGGTGTGCTCAGAATCC-3	NM_204524
*IL-6*	F:5-AAATCCCTCCTCGCCAATCT-3 R:5-CCCTCACGGTCTTCTCCATAAA-3	HM179640
*RELA*	F:5-CAGCCCATCTATGACAACCG-3 R:5-CAGCCCAGAAACGAACCTC-3	D13721
*NFKB1*	F:5-GAAGGAATCGTACCGGGAACA-3 R:5-CTCAGAGGGCCTTGTGACAGTAA-3	NM_205134
*MyD88*	F:5-CCTGGCTGTGCCTTCGGA-3 R:5-TCACCAAGTGCTGGATGCTA-3	NM_001030962
*TLR* *4*	F:5-TTCAGAACGGACTCTTGAGTGG-3 R:5-CAACCGAATAGTGGTGACGTTG-3	NM_001030693
*Occludin*	F:5-GAGCCCAGACTACCAAAGCAA-3 R:5-GCTTGATGTGGAAGAGCTTGTTG-3	NM_205128.1
*ZO-1*	F:5-CCGCAGTCGTTCACGATCT-3 R:5-GGAGAATGTCTGGAATGGTCTGA-3	XM_015278975.2
*JAM2*	F:5-AGCCTCAAATGGGATTGGATT-3 R:5-CATCAACTTGCATTCGCTTCA-3	XM_046940221
*MUC2*	F:5-GCCTGCCCAGGAAATCAAG-3 R:5-CGACAAGTTTGCTGGCACAT-3	XM_040701654

Abbreviations: F = forward primer, R = reverse primer. Nuclear factor erythroid 2-related factor 2 (*Nrf2*), glutathione peroxidase (*GPX*), catalase (*CAT*), superoxide dismutase (*SOD*), heme oxygenase-1 (*HO-1*), interleukin-1β (*IL-1β*), interleukin-6 (*IL-6*), interleukin-10 (*IL-10*), Toll-like receptor 4 (*TLR4*), myeloid differentiation factor 88 (*MyD88*), nuclear factor-κB p50 (*NFKB1*), nuclear factor-κB p65 (*RELA*), zonula occludens protein 1 (*ZO-1*), junctional adhesion molecule 2 (*JAM2*), mucin 2 (*MUC2*).

**Table 3:** The growth performance of broilers as affected by dietary BS and COS supplementation.

Items	Groups						SEM	*p*-Value
	CON	BS1	BS2	COS	MIX1	MIX2		
BW (g)								
d 21	947.31 ^a^	955.73 ^a^	971.12 ^b^	976.23 ^b^	988.29 ^b^	976.63 ^b^	7.85	<0.01
d 42	2716.77	2731.67	2726.79	2704.73	2788.12	2775.91	46.73	0.42
ADG (g/d)								
d 1–21	42.92 ^a^	43.32 ^a^	44.05 ^b^	44.06 ^b^	44.87 ^b^	44.32 ^b^	0.37	<0.01
d 22–42	84.26	84.57	83.60	82.55	85.71	85.68	2.21	0.69
d 1–42	63.59	63.95	63.83	63.30	65.29	65.00	1.11	0.42
ADFI (g/d)								
d 1–21	59.20 ^ab^	62.26 ^b^	61.26 ^b^	62.20 ^b^	62.02 ^b^	59.95 ^a^	0.65	<0.01
d 22–42	167.39 ^a^	172.35 ^b^	166.71 ^a^	173.91 ^b^	177.48 ^b^	168.29 ^a^	1.26	<0.01
d 1–42	114.78 ^a^	118.46 ^ab^	115.29 ^a^	119.37 ^b^	121.16 ^b^	113.91 ^a^	1.04	<0.01
FCR								
d 1–21	0.73 ^b^	0.70 ^a^	0.72 ^ab^	0.71 ^ab^	0.72 ^ab^	0.78 ^c^	0.01	<0.01
d 22–42	0.50	0.49	0.50	0.47	0.48	0.51	0.01	0.16
d 1–42	0.54 ^ab^	0.55 ^b^	0.55 ^b^	0.53 ^a^	0.54 ^ab^	0.57 ^c^	0.01	0.01

Note: BW, body weight; ADG, average daily gain; ADFI, average daily feed intake; FCR, feed conversion ratio. CON = fed basal diet; BS1 = fed basal diet + 5 × 10^9^ CFU/kg BS; BS2 = fed basal diet + 6 × 10^9^ CFU/kg BS; COS = fed basal diet + 125 mg/kg COS; MIX1 = fed basal diet + 5 × 10^9^ CFU/kg BS + 125 mg/kg COS; MIX2 = fed basal diet + 6 × 10^9^ CFU/kg BS + 125 mg/kg COS. Within a row, means bearing different superscript letters differ significantly (*p* < 0.05).

**Table 4:** The effects of BS and COS supplementation on serum antioxidant capacity of broilers.

Items				Groups			SEM	*p*-Value
	CON	BS1	BS2	COS	MIX1	MIX2		
CAT (U/mL)	2.35	2.60	2.41	2.50	2.31	3.17	0.35	0.17
SOD (U/mL)	140.04	124.41	139.41	146.47	139.22	147.91	10.45	0.30
GSH (μmol/mL)	1.92	2.08	1.89	2.05	2.62	2.61	0.38	0.21
GSH-Px (U/mL)	1169.31	1107.59	1114.74	1131.35	1112.48	1221.78	66.86	0.50
T-AOC (U/mL)	11.57	10.98	13.22	12.62	10.98	12.27	1.27	0.42
MDA (mmol/mL)	2.17 ^b^	2.09 ^b^	2.13 ^b^	2.09 ^b^	2.02 ^b^	1.76 ^a^	0.13	0.04

Note: CON = fed basal diet; BS1 = fed basal diet + 5 × 10^9^ CFU/kg BS; BS2 = fed basal diet + 6 × 10^9^ CFU/kg BS; COS = fed basal diet + 125 mg/kg COS; MIX1 = fed basal diet + 5 × 10^9^ CFU/kg BS + 125 mg/kg COS; MIX2 = fed basal diet + 6 × 10^9^ CFU/kg BS + 125 mg/kg COS. Within a row, means bearing different superscript letters differ significantly (*p* < 0.05).

**Table 5:** The effects of BS and COS supplementation on hepatic antioxidant capacity of broilers.

Items				Groups			SEM	*p*-Value
	CON	BS1	BS2	COS	MIX1	MIX2		
CAT (U/mg)	5.26 ^b^	3.35 ^a^	5.11 ^b^	5.76 ^b^	5.94 ^b^	5.70 ^b^	0.77	0.03
SOD (U/mg)	134.94	116.24	133.6	135.93	144.97	110.78	15.10	0.22
GSH (μmol/mg)	5.58 ^b^	3.34 ^a^	5.27 ^b^	5.17 ^b^	7.64 ^c^	6.62 ^bc^	0.97	<0.01
GSH-Px (U/mg)	99.27 ^bc^	61.79 ^a^	96.02 ^bc^	98.93 ^bc^	117.10 ^c^	107.58 ^c^	14.13	0.01
T-AOC (U/mg)	26.45 ^b^	16.41 ^a^	30.55 ^bc^	24.47 ^ab^	35.16 ^bc^	28.34 ^bc^	4.32	0.01
MDA (mmol/mg)	8.76 ^bc^	5.26 ^a^	8.51 ^bc^	9.09 ^bc^	10.63 ^c^	9.45 ^bc^	1.20	<0.01

Note: CON = fed basal diet; BS1 = fed basal diet + 5 × 10^9^ CFU/kg BS; BS2 = fed basal diet + 6 × 10^9^ CFU/kg BS; COS = fed basal diet + 125 mg/kg COS; MIX1 = fed basal diet + 5 × 10^9^ CFU/kg BS + 125 mg/kg COS; MIX2 = fed basal diet + 6 × 10^9^ CFU/kg BS + 125 mg/kg COS. Within a row, means bearing different superscript letters differ significantly (*p* < 0.05).

**Table 6:** The effects of BS and COS supplementation on jejunal antioxidant capacity of broilers.

Items				Groups			SEM	*p*-Value
	CON	BS1	BS2	COS	MIX1	MIX2		
CAT (U/mg)	2.30	2.13	2.00	2.50	2.39	2.74	0.30	0.19
SOD (U/mg)	71.84	72.30	70.52	64.88	66.8	69.58	6.39	0.83
GSH (μmol/mg)	1.44	1.34	1.23	1.45	1.32	1.44	0.22	0.89
GSH-Px (U/mg)	38.53	47.82	39.54	49.97	42.95	50.49	5.51	0.14
T-AOC (U/mg)	6.38	6.17	5.29	6.60	7.14	7.59	0.87	0.16
MDA (mmol/mg)	2.14	1.66	1.71	1.76	1.82	2.12	0.22	0.13

Note: CON = fed basal diet; BS1 = fed basal diet + 5 × 10^9^ CFU/kg BS; BS2 = fed basal diet + 6 × 10^9^ CFU/kg BS; COS = fed basal diet + 125 mg/kg COS; MIX1 = fed basal diet + 5 × 10^9^ CFU/kg BS + 125 mg/kg COS; MIX2 = fed basal diet + 6 × 10^9^ CFU/kg BS + 125 mg/kg COS.

**Table 7:** The effects of BS and COS supplementation on the expression of hepatic antioxidant-related genes in broilers.

Items				Groups			SEM	*p*-Value
	CON	BS1	BS2	COS	MIX1	MIX2		
*CAT*	0.45 ^a^	2.43 ^c^	1.59 ^b^	1.28 ^b^	1.93 ^bc^	1.49 ^b^	0.38	<0.01
*GPX*	0.74 ^a^	1.98 ^b^	1.62 ^ab^	2.08 ^c^	2.62 ^c^	1.12 ^a^	0.34	<0.01
*Nrf2*	0.66 ^a^	1.72 ^b^	1.92 ^b^	0.94 ^a^	1.54 ^ab^	1.10 ^a^	0.32	0.01
*SOD*	0.77 ^a^	1.48 ^b^	1.45 ^b^	1.45 ^b^	2.02 ^c^	1.33 ^b^	0.34	0.04
*HO-1*	0.76 ^a^	1.34 ^b^	1.75 ^bc^	1.29 ^b^	2.10 ^c^	1.54 ^b^	0.17	<0.01

Note: CON = fed basal diet; BS1 = fed basal diet + 5 × 10^9^ CFU/kg BS; BS2 = fed basal diet + 6 × 10^9^ CFU/kg BS; COS = fed basal diet + 125 mg/kg COS; MIX1 = fed basal diet + 5 × 10^9^ CFU/kg BS + 125 mg/kg COS; MIX2 = fed basal diet + 6 × 10^9^ CFU/kg BS + 125 mg/kg COS. Abbreviations: catalase (*CAT*), glutathione peroxidase (*GPX*), Nuclear factor erythroid 2-related factor 2 (*Nrf2*), superoxide dismutase (*SOD*), heme oxygenase-1 (*HO-1*). Within a row, means bearing different superscript letters differ significantly (*p* < 0.05).

**Table 8:** The effects of BS and COS supplementation on the expression of antioxidant-related genes in the jejunum of broilers.

Items				Groups			SEM	*p*-Value
	CON	BS1	BS2	COS	MIX1	MIX2		
*CAT*	0.81 ^a^	1.05 ^a^	2.09 ^c^	1.62 ^b^	1.57 ^b^	1.39 ^ab^	0.31	0.01
*GPX*	1.12	1.12	1.14	1.40	0.93	1.66	0.26	0.12
*Nrf2*	1.02 ^a^	1.65 ^b^	1.30	1.03 ^a^	1.47 ^b^	0.99 ^a^	0.15	<0.01
*SOD*	2.08 ^a^	2.21 ^a^	1.81 ^a^	3.92 ^b^	7.72 ^c^	3.74 ^b^	0.19	<0.01
*HO-1*	0.92	1.57	1.07	1.50	1.38	1.22	0.23	0.09

Note: CON = fed basal diet; BS1 = fed basal diet + 5 × 10^9^ CFU/kg BS; BS2 = fed basal diet + 6 × 10^9^ CFU/kg BS; COS = fed basal diet + 125 mg/kg COS; MIX1 = fed basal diet + 5 × 10^9^ CFU/kg BS + 125 mg/kg COS; MIX2 = fed basal diet + 6 × 10^9^ CFU/kg BS + 125 mg/kg COS. Abbreviations: catalase (*CAT*), glutathione peroxidase (*GPX*), Nuclear factor erythroid 2-related factor 2 (*Nrf2*), superoxide dismutase (*SOD*), heme oxygenase-1 (*HO-1*). Within a row, means bearing different superscript letters differ significantly (*p* < 0.05).

**Table 9:** The effects of BS and COS supplementation on serum immune indices of broilers.

Items				Groups			SEM	*p*-Value
	CON	BS1	BS2	COS	MIX1	MIX2		
IFN-γ	176.33	156.90	179.45	178.31	161.46	159.66	11.86	0.21
IL-1β	19.91 ^a^	29.19 ^b^	24.66 ^ab^	24.25 ^ab^	30.53 ^b^	30.09 ^b^	2.37	<0.01
IL-2	263.08	277.80	283.39	279.69	232.59	267.36	19.62	0.14
IL-4	41.85 ^a^	60.76 ^b^	56.29 ^ab^	53.22 ^ab^	68.37 ^b^	62.58 ^b^	6.41	0.01
IL-6	129.32	139.15	128.57	127.53	111.88	121.95	9.08	0.11
IL-10	120.65 ^b^	135.89 ^c^	117.88 ^b^	102.57 ^b^	84.22 ^a^	136.11 ^c^	7.08	0.11
TNF-α	123.98 ^b^	124.64 ^b^	124.75 ^b^	122.82 ^b^	97.26 ^a^	120.71 ^b^	5.61	<0.01

Note: CON = fed basal diet; BS1 = fed basal diet + 5 × 10^9^ CFU/kg BS; BS2 = fed basal diet + 6 × 10^9^ CFU/kg BS; COS = fed basal diet + 125 mg/kg COS; MIX1 = fed basal diet + 5 × 10^9^ CFU/kg BS + 125 mg/kg COS; MIX2 = fed basal diet + 6 × 10^9^ CFU/kg BS + 125 mg/kg COS. Within a row, means bearing different superscript letters differ significantly (*p* < 0.05).

**Table 10:** The effects of BS and COS supplementation on hepatic immune indices of broilers.

Items				Groups			SEM	*p*-Value
	CON	BS1	BS2	COS	MIX1	MIX2		
IFN-γ	11.25	6.92	9.68	10.79	13.91	11.28	2.78	0.27
IL-1β	14.01 ^a^	23.89 ^b^	17.77	23.10 ^b^	21.37 ^b^	19.42 ^b^	2.73	0.01
IL-2	27.36 ^b^	14.44 ^a^	21.55 ^b^	24.66 ^b^	29.87 ^b^	25.77 ^b^	3.25	<0.01
IL-4	37.21 ^a^	57.26 ^c^	38.53 ^a^	47.30 ^b^	49.32 ^b^	49.29 ^b^	5.39	0.01
IL-6	11.86 ^b^	6.84 ^a^	10.36 ^b^	11.29 ^b^	13.70 ^b^	11.89 ^b^	1.44	<0.01
IL-10	8.65	5.60	8.46	8.48	10.63	9.28	1.54	0.07
TNF-α	9.14	5.39	7.78	10.37	10.42	9.58	1.78	0.07

Note: CON = fed basal diet; BS1 = fed basal diet + 5 × 10^9^ CFU/kg BS; BS2 = fed basal diet + 6 × 10^9^ CFU/kg BS; COS = fed basal diet + 125 mg/kg COS; MIX1 = fed basal diet + 5 × 10^9^ CFU/kg BS + 125 mg/kg COS; MIX2 = fed basal diet + 6 × 10^9^ CFU/kg BS + 125 mg/kg COS. Within a row, means bearing different superscript letters differ significantly (*p* < 0.05).

**Table 11:** The effects of BS and COS supplementation on jejunal immune indices of broilers.

Items				Groups			SEM	*p*-Value
	CON	BS1	BS2	COS	MIX1	MIX2		
IFN-γ	13.69	24.70	16.96	20.16	18.15	16.33	3.80	0.11
sIgA	326.99	393.36	305.57	370.82	380.56	413.33	41.18	0.11
IL-1β	11.23 ^b^	6.45 ^a^	10.23 ^b^	11.04 ^b^	12.88 ^b^	11.33 ^b^	1.56	0.01
IL-2	32.20 ^a^	53.87 ^b^	41.93 ^ab^	50.81 ^b^	51.29 ^b^	50.47 ^b^	5.45	<0.01
IL-4	24.82 ^b^	14.93 ^a^	21.12 ^b^	24.13 ^b^	29.85 ^b^	25.23 ^b^	3.44	0.01
IL-6	14.95 ^a^	24.66 ^b^	19.04	23.04 ^b^	22.10 ^b^	22.17 ^b^	2.57	0.01
IL-10	11.36 ^a^	20.29 ^b^	14.71	20.02 ^b^	18.98 ^b^	15.08	2.87	0.02
TNF-α	8.65 ^a^	19.04 ^b^	12.91	14.73 ^b^	15.66 ^b^	16.01 ^b^	3.00	0.03

Note: CON = fed basal diet; BS1 = fed basal diet + 5 × 10^9^ CFU/kg BS; BS2 = fed basal diet + 6 × 10^9^ CFU/kg BS; COS = fed basal diet + 125 mg/kg COS; MIX1 = fed basal diet + 5 × 10^9^ CFU/kg BS + 125 mg/kg COS; MIX2 = fed basal diet + 6 × 10^9^ CFU/kg BS + 125 mg/kg COS. Within a row, means bearing different superscript letters differ significantly.

**Table 12:** The effects of BS and COS supplementation on the expression of hepatic immune-related genes in broilers.

Items				Groups			SEM	*p*-Value
	CON	BS1	BS2	COS	MIX1	MIX2		
*IL-1β*	0.48 ^a^	1.68 ^b^	1.51 ^b^	1.95 ^c^	2.30 ^c^	1.54 ^b^	0.27	<0.01
*IL-6*	0.40 ^a^	2.23 ^b^	2.35 ^b^	1.60 ^b^	3.30 ^c^	1.68 ^b^	0.31	<0.01
*IL-10*	0.72 ^b^	2.66 ^c^	0.32 ^a^	0.09 ^a^	0.22 ^a^	0.16 ^a^	0.17	<0.01
*TLR4*	0.67 ^a^	1.23 ^a^	2.35 ^c^	1.80 ^b^	1.73 ^b^	1.63 ^b^	0.28	0.01
*MyD88*	0.68 ^a^	1.41 ^b^	1.46 ^b^	1.02 ^ab^	1.24 ^b^	1.39 ^b^	0.19	<0.01
*NFKB1*	0.52 ^a^	1.22 ^b^	1.45 ^b^	0.76 ^a^	1.25 ^b^	0.85 ^a^	0.21	<0.01
*RELA*	0.58 ^a^	1.55 ^b^	1.70 ^bc^	0.96 ^a^	2.11 ^c^	1.40 ^b^	0.41	<0.01

Note: CON = fed basal diet; BS1 = fed basal diet + 5 × 10^9^ CFU/kg BS; BS2 = fed basal diet + 6 × 10^9^ CFU/kg BS; COS = fed basal diet + 125 mg/kg COS; MIX1 = fed basal diet + 5 × 10^9^ CFU/kg BS + 125 mg/kg COS; MIX2 = fed basal diet + 6 × 10^9^ CFU/kg BS + 125 mg/kg COS. Abbreviations: interleukin-1β (*IL-1β*), interleukin-6 (*IL-6*), interleukin-10 (*IL-10*), Toll-like receptor 4 (*TLR4*), myeloid differentiation factor 88 (*MyD88*), nuclear factor-κB p50 (*NFKB1*), nuclear factor-κB p65 (*RELA*). Within a row, means bearing different superscript letters differ significantly (*p* < 0.05).

**Table 13:** The effects of BS and COS supplementation on the expression of jejunal immune-related genes in broilers.

Items				Groups			SEM	*p*-Value
	CON	BS1	BS2	COS	MIX1	MIX2		
*IL-1β*	1.02 ^b^	1.12 ^ab^	0.55 ^b^	0.41 ^a^	1.08 ^b^	0.23 ^a^	0.30	0.03
*IL-6*	1.40 ^a^	1.26 ^a^	1.48 ^b^	1.14 ^a^	1.68 ^b^	1.01 ^a^	0.19	0.03
*IL-10*	0.05 ^a^	0.93 ^c^	0.94 ^c^	0.22 ^b^	0.10 ^a^	0.48 ^b^	0.41	<0.01
*TLR4*	0.93	0.98	0.90	0.93	0.82	0.66	0.27	0.86
*MyD88*	0.19 ^a^	0.11 ^a^	0.25 ^b^	0.27 ^b^	0.15 ^a^	0.17 ^a^	0.14	0.02
*NFKB1*	0.94 ^a^	1.12 ^a^	0.97 ^a^	1.32 ^ab^	1.42 ^b^	1.25 ^ab^	0.14	0.02
*RELA*	1.02 ^ab^	1.50 ^ab^	2.20 ^b^	0.21 ^a^	5.09 ^c^	0.40 ^a^	0.90	<0.01

Note: CON = fed basal diet; BS1 = fed basal diet + 5 × 10^9^ CFU/kg BS; BS2 = fed basal diet + 6 × 10^9^ CFU/kg BS; COS = fed basal diet + 125 mg/kg COS; MIX1 = fed basal diet + 5 × 10^9^ CFU/kg BS + 125 mg/kg COS; MIX2 = fed basal diet + 6 × 10^9^ CFU/kg BS + 125 mg/kg COS. Abbreviations: interleukin-1β (*IL-1β*), interleukin-6 (*IL-6*), interleukin-10 (*IL-10*), Toll-like receptor 4 (*TLR4*), myeloid differentiation factor 88 (*MyD88*), nuclear factor-κB p50 (*NFKB1*), nuclear factor-κB p65 (*RELA*). Within a row, means bearing different superscript letters differ significantly.

**Table 14:** The effects of BS and COS supplementation on the intestinal barrier function of broilers.

Items				Groups			SEM	*p*-Value
	CON	BS1	BS2	COS	MIX1	MIX2		
*JAM2*	0.94 ^b^	1.10 ^b^	0.94 ^b^	1.54 ^c^	1.41 ^c^	0.56 ^a^	0.26	0.02
*Occludin*	1.51 ^a^	1.91 ^b^	0.89 ^a^	1.05 ^a^	1.88 ^b^	2.60 ^c^	0.34	<0.01
*ZO-1*	0.85 ^a^	0.67 ^a^	0.96 ^ab^	1.18 ^b^	0.91 ^ab^	0.92 ^ab^	0.13	0.03
*MUC2*	1.02	0.94	0.73	0.67	0.87	0.44	0.38	0.69

Note: CON = fed basal diet; BS1 = fed basal diet + 5 × 10^9^ CFU/kg BS; BS2 = fed basal diet + 6 × 10^9^ CFU/kg BS; COS = fed basal diet + 125 mg/kg COS; MIX1 = fed basal diet + 5 × 10^9^ CFU/kg BS + 125 mg/kg COS; MIX2 = fed basal diet + 6 × 10^9^ CFU/kg BS + 125 mg/kg COS. Abbreviations: junctional adhesion molecule 2 (*JAM2*), zonula occludens protein 1 (*ZO-1*), mucin 2 (*MUC2*). Within a row, means bearing different superscript letters differ significantly (*p* < 0.05).

## Data Availability

The original contributions presented in this study are included in the article material.
